# Supplementary material for: Association between intrinsic disorder and serine/threonine phosphorylation in Mycobacterium tuberculosis
Source: PeerJ. 2015 Jan 8;3:e724. doi: 10.7717/peerj.724 (PMC4304846; doi:10.7717/peerj.724)
Supplement: Figure S1 [file peerj-03-724-s001.docx]

**Supplemental Figure 1. Frequency distribution of relative evolutionary rate of pS/T.**
