# Supplementary material for: Association between intrinsic disorder and serine/threonine phosphorylation in Mycobacterium tuberculosis
Source: PeerJ. 2015 Jan 8;3:e724. doi: 10.7717/peerj.724 (PMC4304846; doi:10.7717/peerj.724)
Supplement: Figure S2 — The phosphorylation was carried out by purified kinases under in vitro conditions on synthetic 13-mer peptides corresponding to in vivo phosphorylation sites (Prisic et al., 2010). Uncorrected Chi-square p values were significant (<0.05) only for PknA. [file peerj-03-724-s002.docx]

**Supplemental Figure 2. Percentage of disordered phosphoacceptors are shown for different kinases.** The phosphorylation was carried out by purified kinases under *in vitro* conditions on synthetic 13-mer peptides corresponding to *in vivo* phosphorylation sites (Prisic et al.). Uncorrected Chi-square *p* values were significant (<0.05) only for PknA.
